# Cytokeratin 19 (CK19) expression by thyroid neoplasms in a Nigerian tertiary health centre

**DOI:** 10.11604/pamj.2023.44.176.37751

**Published:** 2023-04-14

**Authors:** Soremekun Ademola Idowu, Olaofe Olaejirinde Olaniyi, Komolafe Akinwumi Oluwole

**Affiliations:** 1 Department Morbid Anatomy and Forensic Medicine, Obafemi Awolowo University Teaching Hospital, Ile-Ife, Osun State, Nigeria

**Keywords:** Thyroid, neoplasm, immunohistochemistry, cytokeratin, antibody

## Abstract

**Introduction:**

despite the observed appreciable sensitivity and specificity of the use of CK19 in predicting thyroid malignancies, there is still a paucity of information about its uses in Nigeria. Current information across the world is also scarce. This study was to review the histopathological diagnosis of thyroid neoplasms seen in Obafemi Awolowo University Teaching Hospital Complex (OAUTHC), Ile Ife, using cytokeratin 19 immunohistochemical marker.

**Methods:**

a retrospective study of fifty-six cases of thyroid neoplasm seen over a period of twenty years was conducted. The tissue samples were subjected to immunohistochemical staining for CK19 using monoclonal antibodies. The expression of the markers on the various thyroid neoplasms was assessed histologically.

**Results:**

the expression of CK19 was significantly higher in malignant thyroid neoplasms compared to benign neoplasms (p<0.05). The sensitivity and specificity for cytokeratin 19 were 90.0% and 75.0% respectively.

**Conclusion:**

diffuse immunohistochemical expression of CK19 is a strong indicator of thyroid malignancy. This biomarker can help in the diagnosis of thyroid neoplasms especially those with incomplete or equivocal histomorphology features.

## Introduction

Cytokeratin 19 is a low-molecular-weight cytokeratin found in a variety of simple or glandular epithelia, both normal and neoplastic [1]. It is encoded in humans by keratin-19 (K19) gene and a type 1 keratin [2]. CK19 consists of pairs of different keratin chains of acidic proteins. Unlike its related family members, it is unpaired with a basic cytokeratin in epithelial cells [2]. The type I cytokeratin is clustered in a region of chromosome 17q12-q21 [2]. It is specifically found in the periderm during embryogenesis, the transiently superficial layer that envelops the developing epidermis [2].

Cytokeratin 19 has been found to be positive in malignancies of the anal region, cholangiocarcinoma, endometrial, breast, hepatoid adenocarcinoma, Paget disease (extramammary), pancreatic ductal, squamous cell and thyroid carcinomas [3]. Thyroid neoplasms are the most common neoplasm of the endocrine glands [4]. The benign neoplasms are far more common than the malignant ones by ratios of 10: 1 in the United States [4], 3: 1 in Addis Ababa, Ethiopia [5] and Zaria, Nigeria [6] and by 4: 1 in Accra-Ghana [7] and Ibadan-Nigeria [8]. However, an increase in the incidence of thyroid carcinoma has been noted in recent years and papillary thyroid carcinoma (PTC) is by far the most common type of thyroid malignancies [9].

Normal thyroid follicular epithelium usually has no detectable or weak focal CK19 staining pattern [1]. Many studies have reported strong and diffuse cytoplasmic patterns of staining in PTC [1]. Its expression in other follicular neoplasms such follicular adenoma (FA) or follicular thyroid carcinoma (FTC) are usually weak and focal [1]. Focal and weak staining can also be seen in chronic lymphocytic thyroiditis [10]. The distribution and intensity of CK19 staining is the most critical aspect in its accurate interpretation [10]. Cytokeratin 19 positivity alone lacks specificity for malignancy but combination of strong and diffuse pattern of positivity is an indication for malignancy especially in PTC [10]. Previous studies have observed the overall sensitivity of CK19 to be 79.3% for malignancy, 82.2% for PTC, and 44.3% for FTC [1] and the specificity of 63.1% [10].

Despite the observed appreciable sensitivity and specificity of the use of CK19 in predicting thyroid malignancies, there is still paucity of information about its uses in Nigeria. Current information across the world is also scarce. Considering the above views, this study aimed at evaluating the usefulness of this marker in our environment. This stands to give a more up-to-date information about CK19. The specific objectives were to describe the frequencies of the various thyroid neoplasms and evaluate them for association with age and sex and to describe the immunohistochemical profiles of all the thyroid neoplasms using anti-cytokeratin 19 antibodies.

## Methods

**Study design and setting:** the retrospective study was done in the Department of Morbid Anatomy and Forensic Medicine, Obafemi Awolowo University Teaching Hospital Complex (OAUTHC), Ile-Ife. The hospital is a tertiary health institution in Ife-Ijesa area of Osun State, Nigeria.

**Participants:** this was a retrospective study of all consecutive neoplastic thyroid lesions that were recorded in the daybook of the Department of Morbid Anatomy and Forensic Medicine OAUTHC, Ile-Ife, from 1^st^ July 1996 to 30^th^ June 2016. The diagnosis of thyroid neoplasms during this period were made using histomorphology features of haematoxylin and eosin (H&E) stained slides alone or with Congo red.

### Variables

**Quantitative variables:** age was the only continuous variable. The ages were classified into ten-year age groups for ease of comparison with similar studies from other parts of the world.

**Qualitative variables:** the sex (male and female) of the patients and histological diagnosis (benign and malignant) were noted and recorded. Other categorical variables assessed were specific histologic diagnosis and CK19 immunostain grade (0, 1+, 2+, 3+, 4+). The cases were reviewed blindly and independently without knowing the original histologic diagnosis. The slides were graded based on the percentage of tumour cells that were positive for the antibodies. The expression of the markers was assessed as follows: 0, no staining or staining of less than 10% of tumour cells; 1+, staining of 10% to 25% of the cells; 2+, staining of 26% to 50% of the cells; 3+, staining of 51% to 75% of the cells and 4+, staining of more than 75% of the tumour cells. Zero grading (0) was regarded as negative, 1+ or 2+ was regarded as focal staining and 3+ or 4+ was regarded as diffuse staining [11].

**Data sources/measurement:** all the tissue block samples were subjected to CK19 antibody (Dako RCK108) irrespective of the initial H&E diagnosis. The method used was heat-induced epitope retrieval (HIER). The sections of the tissue specimen were fixed on charged slides. The slides were immersed in citrate buffer (pH=6.0) diluted to 1: 10 with distilled water and placed in a microwave set at 50°C with gradual increment in temperature at 10 minutes interval until 90°C is attained. The slides were then removed and placed in fresh citrate and allowed to cool for 20 minutes in cold water. They were then rinsed in Phosphate Buffer Solution (PBS). Positive and negatives controls were used to assess the integrity of the biochemical reactions for each batch. The positive and negative controls for each antibody were processed along with each batch of slides. The positive control used was skin tissue as specified in the manufacturer´s manual. The slides were placed in a humidity chamber, 3% hydrogen peroxide was added to each tissue for 10 minutes to block endogenous peroxidase and then the slides were rinsed in 0.1% PBS. The specimens were then incubated for one hour with 40-130 μl of an appropriately characterized and diluted Dako mouse ant-CK19 antibody (depending on the surface area of the tissue) at 60°C for one hour. This was followed by incubation with an undiluted labelled polymer Horse Radish Peroxidase (HRP) and conjugated anti-mouse secondary antibody for 30 minutes. The slides were then rinsed with PBS and the areas surrounding the tissue were wiped dry and 1ml of diaminobenzidine solution (catalogue number TA-125-QHDX) was added to cover the specimen. These were incubated in the humidity chamber for 15 minutes. The counterstain used was haematoxylin.

**Study size and bias:** all consecutive cases seen were considered for the study. No sampling was done. The request cards and paraffin embedded tissue blocks of patients with histological diagnosis of thyroid neoplasms were retrieved and studied. Neoplastic thyroid lesions with insufficient embedded tissue or whose slides and blocks could not be retrieved were excluded from the study. In addition, all neoplastic thyroid cases which the biodata of the patients could not be found were also excluded.

**Statistical methods:** the data was analyzed using SPSS 20 and presented using frequency tables, descriptive and comparative statistics. Chi square test was used to compare for non-quantitative and categorical variables. Difference of means of ages was assessed using independent sample t-test. The level of statistical significance was set at p-value: <0.05. Sensitivity, specificity, positive predictive value, and negative predictive value were then calculated.

**Ethical consideration:** approval for the study was obtained from the Ethics and Research Committee of Obafemi Awolowo University Teaching Hospital, Ile-Ife.

## Results

**Participants:** four hundred and ninety-seven (1.58% of the total specimens received during the period of study) thyroid specimens were received within the 20-year period and 80 (16.1% of the total thyroid cases) of the thyroid specimens were thyroid neoplasms. Twenty-four (30%) of these cases were excluded from the study because of inability to retrieve blocks and insufficient amount of tissue in the paraffin embedded blocks to make diagnosis. Fifty-six (70%) neoplastic thyroid cases were used in this study.

**Descriptive data:** sixteen (28.6%) of the cases used in the study were benign and forty (71.4%) cases were malignant. The ratio of the benign thyroid neoplasms to malignant ones was 1: 2.5. The number of males among the neoplastic cases was fifteen (26.8%) and the number of females was forty-one (73.2%). The ratio of males to females among the cases studied was 1: 2.7. Thirteen of the benign thyroid cases were females (81.2%) and three (18.8%) were males. The ratio of female to male for the benign thyroid neoplasm was 4.3: 1. Table 1 shows sex distribution among specific benign and malignant thyroid neoplasms and the frequency distribution of the various histological types in both sexes. Among the forty-one females in this study, twenty-eight (68.3%) had malignant thyroid lesions and thirteen (31.7%) had benign ones. The ratio of females with malignant neoplasm to the ones with benign neoplasm was 2.2: 1. Out of the fifteen (80.0%) males in this study, twelve had malignant neoplastic conditions and three (20.0%) had benign lesions. The number of males with malignant neoplasm was significantly higher when compared to benign neoplasm. The ratio was 4: 1. There was no significant association between sex and the nature (benign/malignant) of the tumour (p=0.38).

**Table 1:** sex distribution of patients with specific benign and malignant thyroid neoplasms

Diagnosis	Sex		Total
	Female	Male	
FA	13 (81.3%)	3 (18.8%)	16 (100.0%)
FTC	2 (33.3%)	4 (66.7%)	6 (100.0%)
Melanoma	1 (100.0%)	0 (0.0%)	1 (100.0%)
MTC	3 (100.0%)	0 (0.0%)	3 (100.0%)
PTC	14 (73.7%)	5 (26.3%)	19 (100.0%)
PTC-FV	8 (72.7%)	3 (27.3%)	11 (100.0%)
Total	41 (73.2%)	15 (26.8%)	56 (100.0%)

FA: follicular adenoma; FTC: follicular thyroid carcinoma; MTC: medullary thyroid carcinoma; PTC-FV: papillary thyroid carcinoma-follicular variant; PTC: papillary thyroid carcinoma

**Figure 1:**
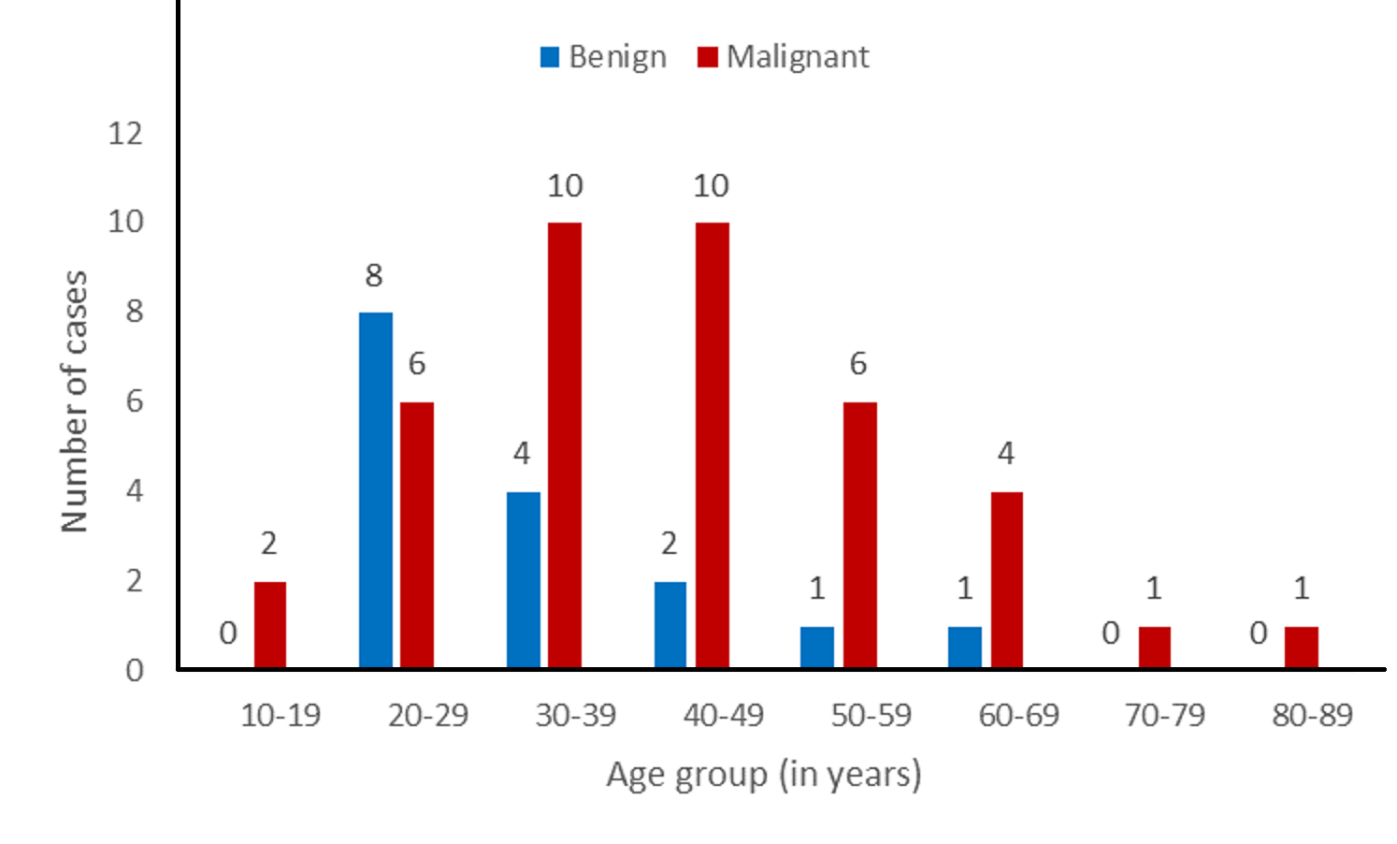
age group distribution of benign and malignant thyroid neoplasms in the study

The ages of the study population ranged from 16 to 83 years. Out of the fifty-six cases of thyroid neoplasms, forty (71.4%) occurred between the ages of 20 to 49 years. The mean age of all the cases was 40 years (39.75 years). Figure 1 shows the age groups of the study population with benign and malignant thyroid neoplasms. Benign thyroid neoplasms were more in the age range of 20-29 years. The mean age of benign thyroid neoplasms is 34 years. The malignant thyroid neoplasms had a peak occurrence between the ages of 30 and 49 years with a mean of 42 years. The mean age of the cases with benign thyroid neoplasms was 8 years lower than that of the patients with malignant thyroid neoplasms.

There were three benign thyroid neoplasms in males and the mean age was 36 years. Benign thyroid neoplasms had a peak between the ages of 20 and 29 years in females with the mean age of 33 years. The mean age of females with benign neoplasms of the thyroid was 3 years lower than that of the male patients. Malignant thyroid neoplasms peak between the ages of 20 and 39 years and has a mean age of 41 years in males. In females, malignant thyroid neoplasms peak between the ages of 40 and 49 years and has a mean of 42 years (42.8 years). There is no significant difference between the mean ages of males and females with malignant thyroid neoplasms in this study (p=0.068).

Follicular adenoma accounted for 100% of the benign neoplasms in the study. Out of all the sixteen benign thyroid neoplasms which were all follicular adenoma, there was one case of follicular adenoma with predominance of Hurthle cell otherwise called Hurthle cell adenoma in a twenty-two-year-old female. The peak occurrence of benign neoplasms in the study was between ages 20 and 29 years. The malignant thyroid neoplasms seen in the study were papillary carcinoma (75.0%), follicular carcinoma (15.0%), medullary carcinoma (7.5%) and melanoma (2.5%). Among the thirty PTCs seen in the study, there were nineteen classic PTC (47.5%) and eleven follicular variants of PTC (27.5%). Papillary thyroid carcinoma (PTC) shows female predominance with male to female ratios of 1: 2.8. The FTC showed male preponderance with a male to female ratio of 2: 1. All the three cases of medullary thyroid carcinoma (MTC) were females. The other malignant case seen was a thyroid mass in a forty-seven-year-old woman which was diagnosed to be melanoma.

**Outcome data and main results:** cytokeratin 19 had cytoplasmic staining pattern. Two (3.6%) of the cases were negative for cytokeratin 19 expression while fifty-four (96.4%) showed varying expression of cytokeratin 19. Fourteen (25%) of the patients expressed cytokeratin 19 focally while forty (71.4%) showed diffuse expression of the antibody. There is a significant statistical relationship between CK19 staining and the neoplastic thyroid samples (p-value: <0.001).

**Figure 2:**
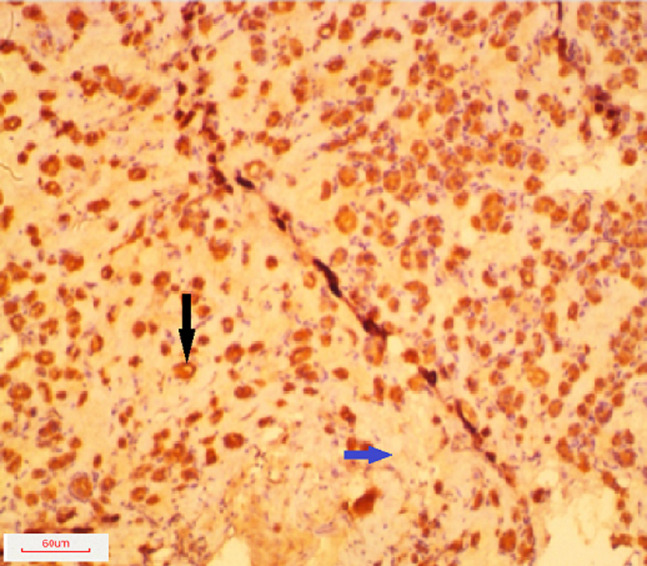
photomicrograph of a patient with follicular adenoma showing micro follicles, oedematous stroma and diffuse staining by CK19 (x200); the black arrow points to a microfollicle and the blue arrow points to the oedematous stroma

Cytokeratin 19 expression was negative in only one (6.3%) of the follicular adenomas but was variably expressed in the remaining fifteen (93.7%) cases. In eleven (68.7%) of the cases, there was focal expression of CK19 and four (25.0%) showed diffuse expression by the tumour cells. Therefore, twelve (75.0%) out of the sixteen follicular adenomas were either negative or focally positive for CK19. The ratio of negative and focal staining of follicular adenoma to diffuse staining was 3: 1. A typical photomicrograph of CK19 staining in follicular adenoma is shown in Figure 2. A considerable number of the follicular adenomas in the study show focal expression of cytokeratin 19. The sensitivity of cytokeratin in distinguishing benign from malignant neoplasms in this study was 90.0%, specificity was 75.0%, positive predictive value was 92.3% and negative predictive value was 76.5%. Table 2 shows expression of cytokeratin 19 in all the study cases.

**Table 2:** expression of cytokeratin 19 in the neoplastic thyroid cases

Diagnosis	Cytokeratin 19			
	Diffuse	Focal	Negative	Total
FA	4 (25.0%)	11 (68.8%)	1 (6.2%)	16 (100.0%)
FTC	5 (83.3%)	1 (16.7%)	0 (0.0%)	6 (100.0%)
Melanoma	0 (0.0%)	0 (0.0%)	1 (100.0%)	1 (100.0%)
MTC	1 (33.3%)	2 (66.7%)	0 (0.0%)	3 (100.0%)
PTC	19 (100.0%)	0 (0.0%)	0 (0.0%)	19 (100.0%)
PTC-FV	11 (100.0%)	0 (0.0%)	0 (0.0%)	11 (100.0%)
TOTAL	40 (71.4%)	14 (25.0%)	2 (3.6%)	56 (100.0%)

FA: follicular adenoma; FTC: follicular thyroid carcinoma; MTC: medullary thyroid carcinoma; PTC-FV: papillary thyroid carcinoma-follicular variant; PTC: papillary thyroid carcinoma

Cytokeratin 19 was expressed in thirty-nine (97.5%) malignant thyroid neoplasms. In three (7.5%) of the cases, there was focal expression of CK19 and thirty-six (90.0%) showed diffuse expression by the tumour cells. Four (10.0%) out of the forty malignant thyroid neoplasms were either negative or focally positive for CK19.

Cytokeratin 19 has diffuse expression in all the thirty PTCs in the study. Cytokeratin 19 was expressed in all the follicular carcinoma in the study. One showed focal CK19 expression and the remaining five were diffusely positive for CK19. There were four other malignant cases in the study. Three were medullary thyroid carcinoma and one was thyroid melanoma. All the three cases expressed cytokeratin 19, two were focal and one was diffuse. The solitary case of melanoma was negative. There was significant association between the pattern of staining and the nature of the tumour (p<0.001).

## Discussion

In this study, fifty-six neoplastic thyroid cases were evaluated with sixteen (28.6%) being benign and forty (71.4%) malignant. The ratio of the benign thyroid neoplasms to malignant ones is 1: 2.5. This agrees with studies done by Ariyibi *et al*. [8], Albasri *et al*. [12] and Adeniji *et al*. [13] in which there were more malignant cases than benign ones and the ratio of benign to malignant neoplasms were 1: 1.3, 1: 10.6 and 1: 1.3 respectively. Albasri *et al*. [12] in Saudi Arabia recorded a higher percentage of malignant cases, 91.4% as against 8.6% for the benign lesions.

There was a female preponderance in our study, the number of females was forty-one (73.2%) and males were fifteen (26.8%). The ratio of females to males was 2.7: 1. This finding is consistent with several works done on thyroid diseases and neoplastic lesions [5,7,8,14]. Among the malignant thyroid neoplasms, female predominance was also striking with 70.0% being females and only 30.0% were males. The ratio of female to male was 2.3: 1. This finding is in harmony with the study done by Der *et al*. [7] in Ghana where 69.7% of the malignant neoplasms were females and 30.3% were males. The female-to-male ratio obtained was same as ours: 2.3: 1. Tsegaye *et al*. [5] and Ariyibi *et al*. [8] reported slightly lower ratios of 1.9: 1 and 1.7: 1 respectively while Ijomone *et al*. [14] and Solomon *et al*. [15] got higher female to male ratios of 4: 1 and 3.7: 1 in that order.

Our study clearly shows higher number of the males (80.0%) have malignant neoplasm than those with the benign ones (20.0%). This finding agrees with the study by Ariyibi *et al*. [8] who also noted higher number of males (69.2%) with malignant thyroid neoplasm compared with benign neoplasm (30.8%). Der *et al*. [7] and Ijomone *et al*. [14] also recorded higher proportions of males with thyroid malignancies compared with benign neoplasms with the following ratios 1.6: 1 and 1.3: 1 respectively. This observation is in support of the known fact in medical parlance that thyroid swellings in males are likely to be malignant than benign hence there is need for thorough sampling.

The malignant thyroid neoplasms, though not as common as other malignancies of the breast, colon, and prostate, is the most common among the endocrine gland malignancies [16,17]. Thyroid carcinomas are far more common than thyroid sarcomas [14]. In this study, only carcinomas were found amongst the malignant thyroid cases. This finding is supported by both local [6,8,13,14,18,19] and foreign [5,7,12] works on thyroid diseases and neoplasms. Ahmed *et al*. [6], Tsegaye *et al*. [5] and Ijomone *et al*. [14] reported all the thyroid malignancies in their study series to be carcinomas. Lawal *et al*. [18] in Ile-Ife, Albasri *et al*. [12] in Saudi Arabia and Ariyibi *et al*. [8] in Ibadan noticed higher percentages of carcinomas in their works but they also found a small number of non-epithelial malignancies like lymphomas and sarcomas. In this study, follicular carcinoma is the second most common primary malignancy of the thyroid accounting for 15.0% of the malignant cases. This observation agrees with the works done in Ahmadu Bello University Teaching Hospital [6] where follicular carcinoma constituted 15.9% of the malignant neoplasms, University of Port Harcourt Teaching Hospital [14] where follicular carcinoma was 30.0% of the malignant thyroid neoplasms and University College Hospital [8] where follicular carcinoma was 32.7% of the malignant lesions.

In the index study, 25.0% of the follicular adenomas showed diffuse expression of cytokeratin 19 by the tumour cells and the remaining 75.0% were either negative or focally positive. The ratio of negative and focal staining of follicular adenoma to diffuse staining was 3: 1. This finding was comparable with that of Park *et al*. [20] that observed 28.6% cytokeratin 19 positivity. Scognamiglio *et al*. [21] got a lower value of 14% and interestingly, all the follicular adenomas were negative for cytokeratin 19 in the study done by Nechifor-Boila *et al*. [11].

In our work, the general sensitivity of CK19 in distinguishing benign from malignant neoplasms was 90.0% and the specificity was 75.0%. The sensitivity value was like that documented by Park *et al*. (90.3%) [20]. However, the specificity value in their study was 83% and higher than the value we got in our study. All the PTCs (100.0%) in our study showed diffused pattern of CK19 expression. This finding agrees with the observation of Scognamiglio *et al*. [21] in New York where they also recorded 100.0% staining of the papillary carcinomas. However, Park *et al*. [20] reported a lower value of 96.7%.

Cytokeratin 19 expression was diffuse in 83.3% of FTCs in our study and this report can be compared with the finding of Saleh *et al*. [22] that got 86.0% positive expression of CK19. The four other malignant cases in the study include three medullary carcinomas and a thyroid melanoma. Cytokeratin 19 staining of MTCs in this study revealed diffuse staining in one (33.3%) of the cases and the other two were focal. This finding contrasts with the observation of Palo *et al*. [23] where the only MTC case used was negative.

It is, however, important to note that owing to the rarity of medullary thyroid carcinoma the number of cases used in these studies including ours are small. A multi-centred study with pooling together of large numbers of MTC cases may give more statistically significant findings. In addition, the antibodies used in these studies are believed to be useful for follicular cell-derived thyroid neoplasms not medullary thyroid carcinoma that has a different histogenesis-the parafollicular cells of the thyroid. The solitary case of melanoma in this study was negative for CK19. This finding further strengthens our understanding that the antibodies used in this study are only useful in differentiating follicular cell-derived neoplasms.

**Limitations:** antigen retrieval may not be adequate in the archival Formalin Fixed Paraffin Embedded (FFPE) tissue blocks used in this study. Although positive and negative controls were used in this study, this cannot completely surmount the challenges of lost antigens because of long-term storage of tissue.

## Conclusion

This study has shown the preponderance of both benign and malignant thyroid neoplasms among females and that thyroid neoplasms in males are likely to be malignant than benign. The mean age of benign thyroid neoplasms has been noted to be about eight years lower than the malignant ones. There is an association between CK19 and the nature of thyroid neoplasms. It is, however, important to note that immunohistochemistry (IHC) is a supplementary procedure that helps in equivocal cases. It is not a replacement for H&E histomorphology interpretation. Interpretation of IHC in thyroid neoplasms also calls for caution because occasional focal positive staining by the immunomarkers used in this study could occur in benign neoplastic cases and very rarely in non-neoplastic lesions. Strong and diffuse staining of tumour cells is the only indicator of malignancy.

### What is known about this topic

- *CK19 is expressed by thyroid cancers in Europe and America;*
- *Focal CK19 positivity is seen in benign tumours*.

### What this study adds

- *CK19 is expressed by thyroid cancers seen in OAUTHC, Ile-Ife;*
- *Focal CK19 positivity is observed in non-malignant thyroid tumours seen in OAUTHC, Ile-Ife*.
